# Emergency Departments as the Health Safety Nets of Society: A Descriptive and Multicenter Analysis of Social Worker Support in the Emergency Room

**DOI:** 10.7759/cureus.3247

**Published:** 2018-09-04

**Authors:** Sasha Selby, Dongmei Wang, Eoin Murray, Eddy Lang

**Affiliations:** 1 Medicine, University of Limerick, Limerick, IRL; 2 Alberta Health Services, University of Calgary, Calgary, CAN; 3 University of Limerick, University Hospital Limerick, Limerick, IRL; 4 Emergency Medicine, University of Calgary, Calgary, CAN

**Keywords:** social work, emergency medicine, safety net, analysis, emergency department (ed), multidisciplinary care

## Abstract

Introduction

Social Work (SW) referrals made in the emergency department (ED) highlight the weaknesses in the existing support system for vulnerable and disadvantaged patients. SW personnel play a pivotal role in some EDs but are not integrated into the team in several jurisdictions. Our objective was to provide a detailed description of the need for SW support in the ED setting by describing SW consultation patterns in an urban ED location.

Methods

A three-year analysis of ED SW referrals made through a network of four acute care hospitals serving a city population of 1.2 million inhabitants where social workers operate from 8 a.m. to 10 p.m. The study design was descriptive reporting proportions. The descriptors of interest were the types of ED patients receiving SW consultations and the reasons for patient referral to the SW Department.

Results

During the study period, there were 46,970 SW consultations, representing 8.02% of the 572,804 patients who visited the ED across Calgary, yielding 42.9 referrals per day to social workers through the ED. Consultations for domestic violence were three times more prevalent for women (6% of referrals). However, domestic violence consultations were still an active issue for men (1.9%). Comparisons by age group yielded illness adjustments (15.3%), discharge planning (31.2%), and legal decision making (23.9%) as the most common reasons for referral of patients over 75 years old; 92.8% of patients over 75 years were admitted following the SW consultation. Reasons for deferral of patients under 30 years of age were illness adjustments (12.2%), discharge planning (16.4 %), and legal decision making (1.4%); 57.3% of patients under 30 years were admitted following the consultation. Addiction/drug use and homelessness were more common in those under the age of 30, comprising 24.1% and 15.4% of the SW referrals, respectively, compared to 1.6% and 0.4% of referrals for those over age 75, respectively.

Conclusions

The demand for SW support is significant and complex in these large urban EDs. However, the impact on patient care and resource use is substantial, and the data indicates that SW integration may be of universal benefit to EDs. Further studies are warranted to accurately characterize the amount and type of SW necessary for optimal patient outcomes and hospital resource use.

## Introduction

Emergency departments (EDs) are those facilities that specialize in acute and urgent care for the local population. EDs often deal with a wide range of illnesses and conditions and may be the first point of care for patients. In addition to the acute management of patients, EDs also serve as a general entry point that links the population (especially those patients with limited access) to health care [1].

In addition to their medical presentations, patients who present in the ED often disclose their social problems, ranging from financial concerns to abuse [2]. Our patient population is aging, which contributes to a significant financial strain on society [3]. Furthermore, elderly patients often present with psychosocial matters that accompany their medical concerns, frequently requiring the intervention of a social worker [4]. In addition to the burden of an aging population, issues of substance abuse and increased illicit drug use land many patients in the ED with significant social problems, which suggests that the role of Social Work (SW) in the ED is expanding [5]. While EDs play an essential safety net role for the population at large, SW staffing varies. Hospital administrators have the difficult task of justifying funding for SW personnel as many hospitals try to limit costs and unnecessary spending. However, research shows that SW personnel address some of the more complex cases seen in an acute care setting and are a vital part of any hospital [6].

Overall, the role of SW in the ED is comprehensive and may involve specific resource mobilization or patient counseling, including functioning as a patient emotional outlet [7]. An ED social worker has many far-reaching roles and is integral in the treatment and management of patients from the ED [2]. Fusenig identified the major roles in ED SW as assessing the psychosocial, social, and medical needs of the patient; working with interpreters and advocating for the patient’s cultural values (as a cultural broker); acting as chemical dependency counselor; acting as liaison for abuse reporting/counseling and providing resources; and acting as a patient advocate for those receive continuing medical treatment.

Although the role of social workers in the ED has been studied and described to some degree in prior research, reported findings are still limited. Thus, further inquiry is warranted as multiple studies suggest that social workers in the ED are often under-utilized, and many physicians in the ED are unsure of how social workers can be used and integrated into patient care and management [8]. The objective of this study was to describe the role and workload of social workers in the ED.

## Materials and methods

We conducted an observational and descriptive study, reporting proportions within our outlined parameters such as presenting concern, length of stay, and discharge diagnosis. Our sample size was not determined prior to the study, but we included any patient who attended an ED in the city of Calgary and was referred to an SW from July 1, 2013, to June 20, 2016. Specifically, the network of the city of Calgary included in this study encompassed four acute care hospitals serving a population of 1.2 million people. Foothills Medical Center (FMC), Peter Lougheed Center (PLC), Rockyview General Hospital (RGH), and South Health Campus (SHC) were the four acute care hospitals included in the study. Only the consultations sought within the three-year period from July 1, 2013, to June 30, 2016, were included in the study.

We analyzed and compiled all of the SW referrals made in the ED using deidentified administrative data extracted from a city-wide electronic health record. The identification of SW consultations (by either a nurse or physician) was obtained through Sunrise Clinical Manager (SCM) (Allscripts, Chicago, Illinois). We used International Statistical Classification of Diseases and Related Health Problems, Version 10 (ICD-10) coding to quantify the consultations obtained through the SCM and compiled variables such as patient demographics, presenting concerns, time and date of arrival, admission rate, and length of stay.

The outcomes of interest were the percentage of ED patients for whom SW consultations were sought, ED length of stay, the most common presenting concerns, and reasons for referral. 

Simple descriptive statistics were also used to analyze the data extracted from SCM using proportions, medians, and interquartile ranges.

The ARECCI (A project ethics community consensus initiative) scoring system is a method of assisting project leaders involved in quality improvement and evaluation projects to evaluate the ethical implications of their studies. Using the ARECCI tool, our study was determined to involve “minimal risk” and was assigned a score of six. A low score indicates a minimal risk to subjects whose data are ascertained from the study [9]. This project is determined to be a quality improvement study on the basis that it outlines the role of SW to generate a better understanding of how different services operate in the ED to allow administrators to more effectively hire staff and provide resources to ensure these services are available in the ED setting.

## Results

Of the approximately 572,804 patients who visited the ED within July 1, 2013, to June 30, 2016 period, SW personnel were consulted 46,970 times, encompassing 8% of all patients assessed, yielding 42 referrals per day. The average age of patients requiring an SW referral was 55 years, and the most frequently referred age was 21 years.

The most common reasons for SW referral for patients over 75 years old were illness adjustments (15%), discharge planning (31%), and legal decision making (23%); and 93% of said patients were admitted after an SW consultation. For patients under 30 years, illness adjustments (12%), discharge planning (16%), and legal decision making (1%) were not as common for referrals to SW; and 57% of patients under 30 were admitted following the consultation. Addiction/drug use and homelessness were more common reasons for SW referrals in those under 30 years old, comprising 24% and 15% of SW referrals compared to 2% and 0.4% of those over age 75 years, respectively. Comparison by sex yielded similar results. However, consultations for domestic violence were three times more prevalent for women (6% of referrals) than men (2%; Tables 1-2).

**Table 1:** Comparison of SW referral reasons by age group Abbreviations: SW, social work; EMS, emergency medical services; ED, emergency department; LOS, length of stay.

Patient Descriptors	Patients < 30 years	Patients aged 30-74	Patients > 75 years	Combined Mean
Mean age (years)	23.7	52.4	84.4	55.2
Incidence of men	52.6%	57.6%	43.4%	53.7%
Arrival by EMS	46.0%	57.8%	77.4%	60.4%
Police arrivals	13.0%	6.0%	1.8%	6.1%
Homeless	15.4%	11.3%	0.4%	9.5%
Illness adjustment	12.2%	18.2%	15.3%	16.7%
Financial concerns	20.5%	25.1%	14.3%	22.0%
Addiction or drug use	24.1%	15.4%	1.6%	13.7%
Discharge planning	16.4%	19.1%	31.2%	21.4%
Violence and assaulted	8.2%	3.9%	0.7%	3.8%
Psychosocial assessments	2.8%	2.1%	1.1%	2.0%
Admitted patients	57.3%	77.3%	92.6%	77.7%
ED LOS (hours)	10.6	12.5	16.0	13.0

**Table 2:** Comparison of SW referral reasons by gender Abbreviations: SW, social work; EMS, emergency medical services; ED, emergency department; LOS, length of stay.

Patient Descriptors	Female Patients	Male Patients	Combined Mean
Mean age (years)	56.5	54.0	55.2
EMS arrivals	59.9%	60.8%	60.4%
Police arrivals	5.9%	6.3%	6.1%
Illness adjustment	16.7%	16.7%	16.7%
Homeless	5.9%	12.6%	9.5%
Violence and assaulted	6.0%	1.9%	3.8%
Addiction or drug use	11.2%	15.8%	13.7%
Legal decision-making	9.4%	6.4%	7.8%
Hospital admissions	75.8%	79.4%	77.7%
Mean ED LOS (hours)	13.1	12.9	13.0
Financial concerns	18.4%	25.2%	22.0%
Psychosocial assessment	2.5%	1.6%	2.0%

Comparison by site yielded some interesting variations—most notably, the percentage of homeless patients with no fixed address that required an SW consultation. RGH had the largest percentage of SW referrals for homeless patients (14%), followed by PLC (11%), FMC (7%) and SHC (6%). The percentage of SW referrals due to addiction/drug use at RGH was 19%, followed by PLC at 13%, SHC at 13%, and FMC at 10%. The percentage of SW referrals for violence and assaults were 6% for RGH, 4% for PLC, 3% for FMC, and 3% for SHC (Table 3).

**Table 3:** Comparison of SW referral reasons by site Abbreviations: FMC, Foothills Medical Center; PLC, Peter Lougheed Center; RGH, Rockyview General Hospital; SHC, South Health Campus; SW, social work; SD, standard deviation; ED, emergency department; LOS, length of stay.

Patient Descriptors	FMC	PLC	RGH	SHC	Total
Number of records	17,708	9,301	13,581	6,379	46,969
Incidence of patients > 65 years	35.1%	29.1%	32.7%	42.1%	34.2%
Incidence of patients > 75 years	21.7%	17.2%	22.4%	28.6%	22.0%
Incidence of patient < 30 years	13.4%	15.5%	16.9%	13.7%	14.9%
Mean age (SD)	56.0 (20.5)	53.0 (20.4)	54.1 (21.9)	58.3 (22.3)	55.2 (21.2)
Number of men (%)	10,252 (57.9%)	5,096 (54.8%)	6,849 (50.4%)	3,042 (47.7%)	25,239 (53.7%)
EMS arrival	67.0%	48.8%	60.3%	59.1%	60.4%
Police arrival	3.0%	7.1%	8.6%	7.9%	6.1%
Illness adjustment	22.4%	16.0%	10.0%	15.9%	16.7%
Discharge planning	23.9%	15.9%	16.6%	32.5%	21.4%
Addiction or drug use	10.1%	13.0%	19.1%	12.9%	13.7%
Homeless	6.6%	10.6%	14.4%	5.7%	9.5%
Violence and assaulted	2.6%	3.7%	5.8%	3%	3.8%
Hospital admissions	90.9%	80.0%	57.4%	81.1%	77.7%
Mean ED LOS (hours)	11.0	12.9	12.9	18.7	13.0

**Figure 1:**
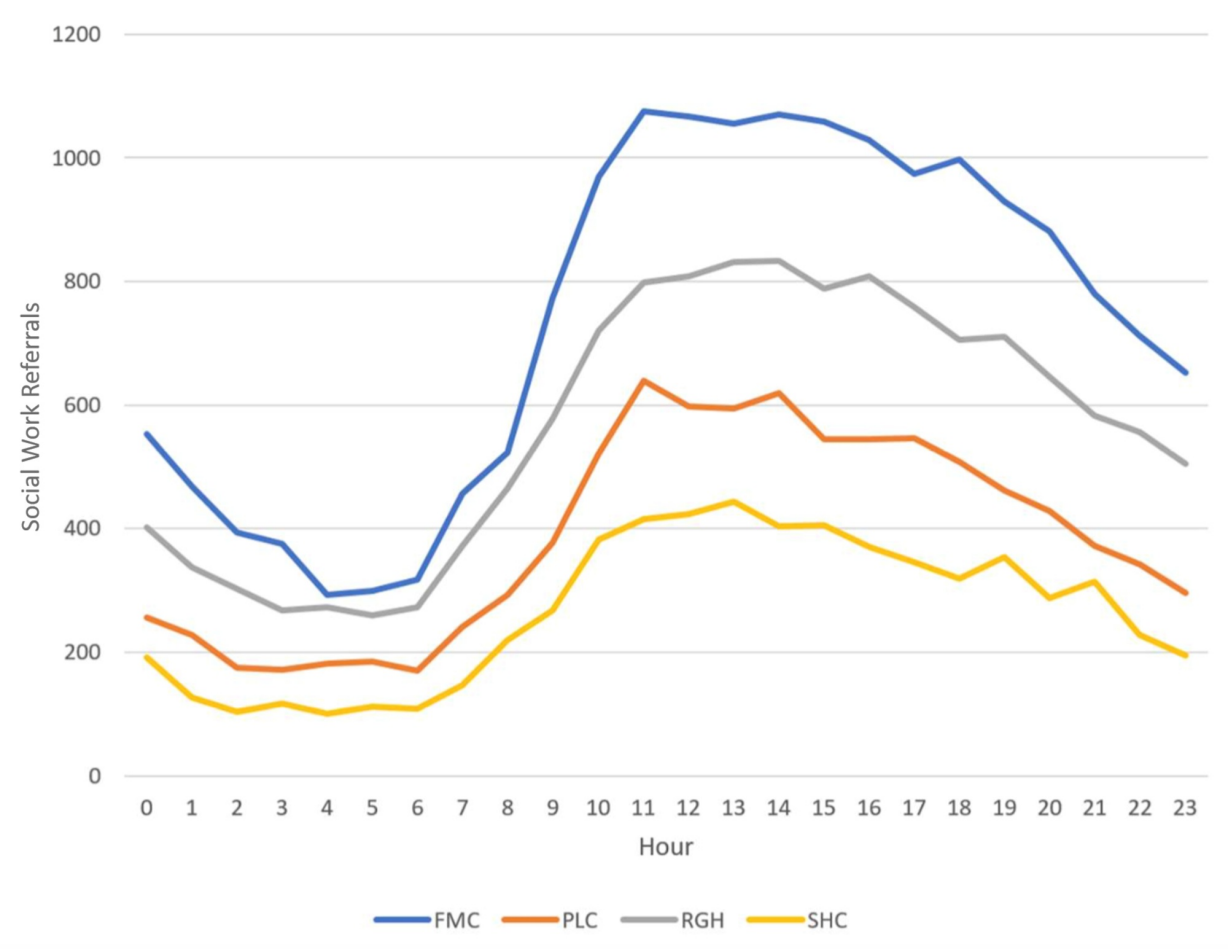
Mean emergency department visits with social work referrals by arriving hour. Abbreviations: FMC, Foothills Medical Center; PLC, Peter Lougheed Center; RGH, Rockyview General Hospital; SHC, South Health Campus

**Figure 2:**
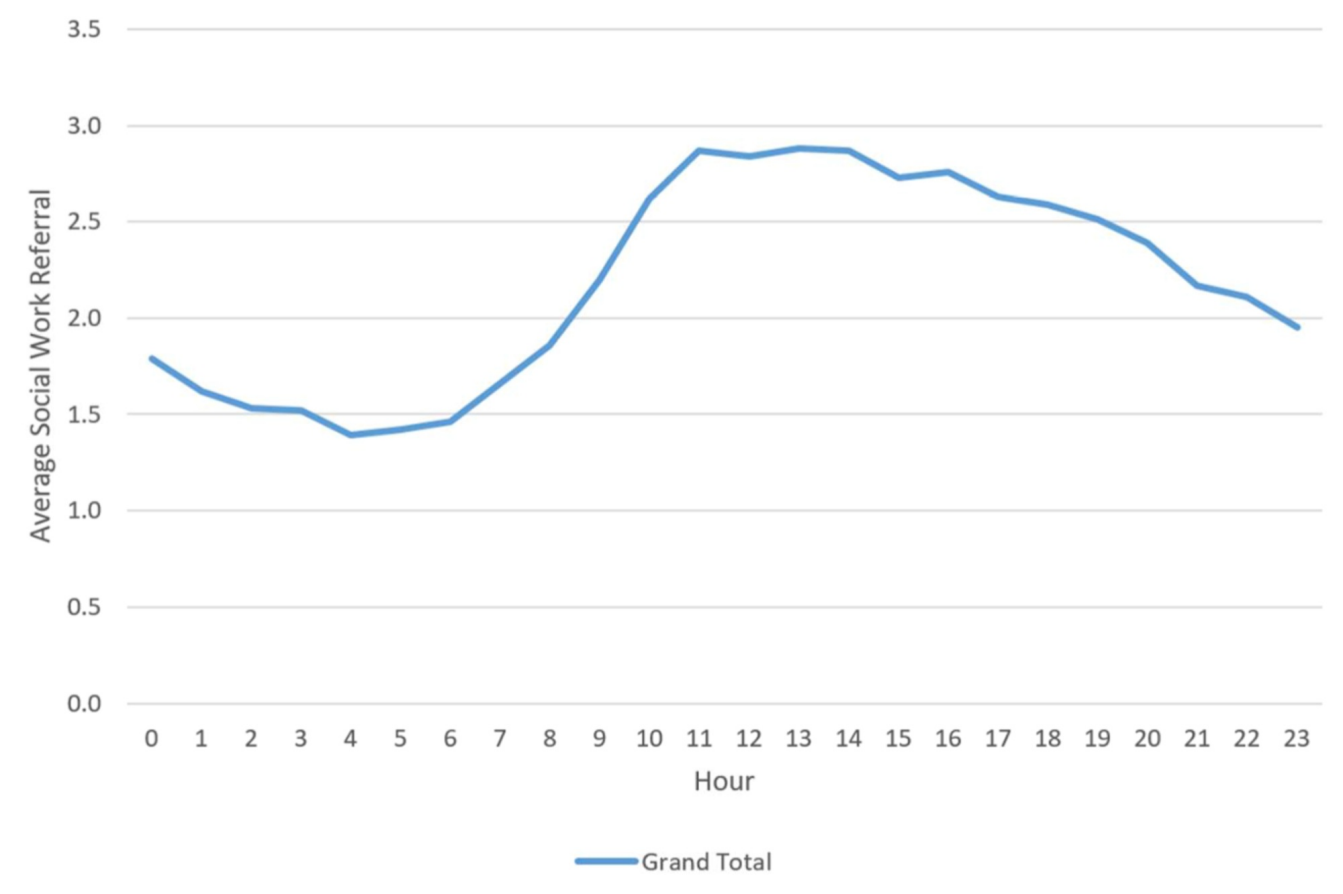
Mean daily visits with social work referrals by hour

No particular month or day of the week denoted any significant variations in referral rates. However, we noted differences relating to the time of arrival of the patient and the number of referrals. However, we noted that the most congested hours of an ED correlated with the data on SW referrals, suggesting that the largest number of SW consultations occur between 10 a.m. and 6 p.m., and the greatest volume occurs from 11 a.m. to 2 p.m. (Figures 1-2).

Given that the most common presenting concern in the ED is shortness of breath (SOB), it was not surprising that SOB was a common reason for SW referrals (8%). Other top presenting concerns for SW referral were abdominal pain (7%), depression or suicidal ideation (6%), and major traumas (4%; Table 4). The most common reasons for consulting an SW involve issues such as financial concerns (17%) discharge planning (17%), illness adjustments (16%), and addiction (11%). Other reasons for referral include resource counseling (9%), care planning (5%), abuse (4%), and psychological assessment (2%).

**Table 4:** Comparison of presenting concerns for SW referrals by site Abbreviations: FMC, Foothills Medical Center; PLC, Peter Lougheed Center; RGH, Rockyview General Hospital; SHC, South Health Campus; ED, emergency department; SW, social work; SOB, shortness of breath.

Presenting concern	FMC	PLC	RGH	SHC	Total
SOB	1,199	1,019	977	572	3,767
Abdominal pain	1,070	735	977	572	3.256
Depression/suicidal thoughts	546	736	1,033	497	2,812
Major trauma, blunt	2,252	68	99	41	2,460
Altered level of consciousness	828	328	676	240	2,072
General weakness	505	346	551	316	1,718
Chest pain (cardiac features)	595	298	333	142	1,368
Substance misuse	273	276	650	156	1,355
Symptoms of stroke	1,175	35	43	52	1,305
Lower extremity injury	436	247	325	274	1,282
Seizures	430	140	351	129	1,050
Bizarre behavior	237	236	385	174	1,032
Vomiting and/or nausea	356	229	289	149	1,023
Confusion	401	163	231	154	949
Localized swelling/redness	221	301	221	95	838

Referrals were made for nearly 150 different conditions of immense variety. Some include substance misuse, fever, cardiac pain, and even simple calluses. This indicates that SW personnel are tasked with consulting on a wide variation of concerns. Approximately 60% of these patients arrived at the ED by emergency medical services. There are significantly more ED SW referrals for the initial concern of SOB than for reasons more commonly associated with the field of SW such as “concern for patient’s welfare” or “situational crisis.”

In keeping with the extensive variety observed in the presenting concerns of those patients referred to SW, the discharge diagnoses from the physicians also demonstrate a similar variety. Mental and behavioral disorders due to the use of alcohol encompass the largest proportion (3%) of diagnoses made at the time of discharge for those patients requiring a SW consultation. The next most prevalent diagnosis associated with a SW referral is sepsis (2%), followed by congestive heart failure (2%), and pneumonia (2.0%; Table 5). There are over 1,000 other listed diagnoses including social, mental, and physical related concerns. This highlights a possible challenge for the SW staff to address such an array of cases, many of which are likely extremely complex.

**Table 5:** Discharge diagnosis code by site Abbreviations: FMC, Foothills Medical Center; PLC, Peter Lougheed Center; RGH, Rockyview General Hospital; SHC, South Health Campus.

Discharge Diagnosis Code	FMC	PLC	RGH	SHC	Totals
Mental and behavioural disorders due to use of alcohol, withdrawal state	375	260	448	204	1,287
Sepsis, unspecified	283	277	215	161	1,036
Congestive heart failure	339	258	241	195	1,033
Mental and behavioral disorders due to use of alcohol, acute intoxication	104	147	589	123	963
Pneumonia, unspecified	303	251	231	177	962
Chronic obstructive pulmonary disease with acute exacerbation, unspecified	202	189	204	138	733
Adjustment disorders	102	170	332	113	717
Unspecified nonorganic psychosis	118	78	345	89	630
Toxic effect of ethanol	118	78	345	89	630
Unspecified dementia	127	128	236	122	613

## Discussion

To encourage quality SW care in the ED setting, it is essential for medical practitioners to better understand and quantify the needs and demands of SW personnel in the ED. Across the four regional hospitals of Calgary, there were a total of 46,970 patients presenting to the ED who were referred for SW consultation between July 1, 2013, and June 30, 2016. Although the number of referrals alone does not give a sufficient picture of the average workload of SW staff, with an average of 43 SW consultations per day, we can establish an objective baseline demand for SW in the ED. To enrich our understanding of SW in the ED, it is imperative that, along with estimating the quantitative number of cases, we are also able to describe, in detail, the types of cases that sought the attention of SW services. Each SW case is unique and can have considerable variations in the amount of work dedicated to the case; this can greatly affect the outcome and follow-up of patient care [7]. A systematic review of the literature from 1950 to 2015 showed that the cases that integrated SW in management provided for the most cost-effective treatment while yielding moderate reductions in overall ED admissions [10].

In general, cases of crisis involving SW may include drug abuse, physical abuse or matters of addiction. Specifically, SW may provide resources and aid in the organization and planning of future detoxification programs or targeted therapy groups. Previous studies have shown that EDs commonly act as a place to seek and coordinate social welfare, especially for people from lower-income classes [11]. Because of this demand for non-emergency services on ED departments, the quality of care, patient flow, and follow-up services can be affected negatively [12]. Many physicians report a lack of competence and training for various conditions such as drug and alcohol abuse compared with the training and expertise of a social worker [13]. SW staff may also manage cases of abuse in adults specifically by organizing a safe plan for the victim and initiating the necessary steps for legal involvement. Notably, our results showed that a greater proportion of women presented with cases of violence or assault, compared to men. However, it is worth noting that men were also greatly affected by violence or assault. Therefore, ED staff should be cognizant of this when involved in sensitive consultations concerning both genders. In cases involving children, SW staff may similarly manage their safety and security and plan future therapy/counseling sessions and housing needs [14]. Especially in vulnerable populations, such as children with traumatic experienced, many ED physicians report lacking the necessary skills or knowledge for properly addressing the psychosocial needs of patients [15]. Though many different demographics may inundate the ED, the key aim is increasing the ease of access and the ability of the EDs to treat a wide variety of issues, both medical and non-medical [16]. 

In this study, the most represented reasons for referral (comprising 69.7% of total referrals) in descending order were financial concerns, discharge planning, illness adjustment, addiction issues, and resource counseling. These results reflect the broad range of long-term planning and care orchestrated by SW personnel. Although financial concerns can relate to all demographics of a population, several studies have shown that the use of the ED is higher among patients from lower socioeconomic groups due to their affordability over ambulatory or primary care settings [1]. Further studies have also suggested that patients from lower socioeconomic groups have longer stays in the hospital and have difficult discharging and post-hospital transitions [17,18]. While our study could not determine if these numbers correlated with socioeconomic status, further research could search for a correlation and, if such a correlation exists, studies could determine how the safety net of SW in the ED can influence these outcomes. Furthermore, the variations between sites regarding issues such as homelessness, addiction and drug use, and violence points to a possible variations in sociodemographic mix of the populations that reside around the respective hospitals. This may yield possible future staffing arrangements for SW services to better address these differing populations by site.

Based on our results, most patients with an SW referral presented with SOB, abdominal pain, depression/suicidal ideation, and major trauma. Even though SOB is a common presenting concern, it is interesting to note in relation to SW as both at-risk and low socioeconomic group patients have been shown to use the ED more frequently for asthma exacerbations, which often present as SOB [19,20]. Depression and suicidal ideation are common in patients of low socioeconomic status, and close follow-up of these patients can improve overall outcomes [21]. The integration of SW personnel intto health care has proved beneficial for reducing the incidence of adverse outcomes such as reduced mortality rates in maternal and neonate populations [22].

We found the average age of patients to be 55.2 years, while the most referred age was 21 years. The mode of 21 can positively skew the distribution curve of the patient population’s age. This may be indicative of an at-risk group that visits the ED composed of both young adults and elderly patients. This reflects recent research that demonstrates that elderly populations [23] and young adults [24] are frequent users of the ED, and that both groups have unique concerns with regards to SW and follow-up care. The variation in patient factors in the various age groups reflects the variety of reasons for SW referral we found, such as the prevalence of addiction or police arrival in younger age groups and illness adjustment and discharge planning in older age groups.

We found no variation between SW referral and the admission volume of the ED. We noted no unusual increase or decrease in the time of day, week or month for SW referrals, with the highest amount of SW referrals seen during routinely high ED volume between 10 a.m. and 6 p.m. These data can provide the ED with guidelines for planning resources and staffing. ED overcrowding can lead to hazardous outcomes for patient care [25], and adequate staff sizes for EDs will help mitigate the risks due to overcrowding. Appropriate staffing allows patients to be suitably cared for and discharged to the appropriate services to decrease possible sentinel and adverse events. Our findings align with those of other studies reporting peak ED times between 10 a.m. and 1 p.m., which may explain the rise in SW referrals from the increased ED volume [26]. A review of the literature from 1990 to 2002 noted that a potential solution for ED overcrowding was multidisciplinary support, such as the integration of SW personnel [27]. 

SW visits can help reduce admissions from frequent ED users through several other methods such as case management, care plans, diversion strategies, and printing out case notes [28]. Individual care plans not only reduce admissions but reduce investigations done on admission as well [29], and case management also seems to be cost-effective while yielding statistically and clinically significant reductions in psychosocial problems compared with usual care [30]. A 2008 study reported that up to 15.7% of ED patient admissions needed the involvement of a social worker, and that use of SW personnel was vital in a cost-containment strategy [6].

Our study was limited by its quantitative focus, and more research on the qualitative side is warranted to give a more accurate assessment of the demands faced by SW personnel in the ED setting. With more comparative and qualitative studies, EDs may be able to increase social workers’ impact and collaboration with other healthcare professionals. Another limitation of our study is the generalizability of the results. Specifically, while this study explored four different acute care hospitals to reflect the different demographics of the City of Calgary, the level to which these hospitals reflect the populations in their respective areas is unknown. The degree of generalizability of the results to other populations elsewhere in Canada is also uncertain, given that Calgary, Alberta, may not be a good representative sample of Canada as a whole.

## Conclusions

Integrating SW into the ED is complex, but the impact on patient care and resource use is substantial and promising. Therefore, careful planning of resources for SW integration into the ED setting is warranted to help meet both, the medical and social care needs of patients. Further studies are warranted to accurately characterize the amount and type of SW necessary for optimal patient outcomes and hospital resource use. 
